# P53-induced miR-1249 inhibits tumor growth, metastasis, and angiogenesis by targeting VEGFA and HMGA2

**DOI:** 10.1038/s41419-018-1188-3

**Published:** 2019-02-12

**Authors:** Xiaoxiang Chen, Kaixuan Zeng, Mu Xu, Xiangxiang Liu, Xiuxiu Hu, Tao Xu, Bangshun He, Yuqin Pan, Huiling Sun, Shukui Wang

**Affiliations:** 1 General Clinical Research Center, Nanjing First Hospital, Nanjing Medical University, 210006 Nanjing, Jiangsu China; 2 Medical College, Southeast University, 210009 Nanjing, Jiangsu China

## Abstract

MicroRNAs (miRNAs) are important class of functional regulators involved in human cancers development, including colorectal cancer (CRC). Exploring aberrantly expressed miRNAs may provide us with new insights into the initiation and development of CRC by functioning as oncogenes or tumor suppressors. The aim of our study is to discover the expression pattern of miR-1249 in CRC and investigate its clinical significance as well as biological role in CRC progression. In our study, we found that miR-1249 was markedly downregulated in CRC tissues and cell lines, and negatively related to pN stage, pM stage, TNM stage, and overall survival (OS). Moreover, we demonstrated that miR-1249 was a direct transcriptional target of P53 and revealed that P53-induced miR-1249 inhibited tumor growth, metastasis and angiogenesis in vitro and vivo. Additionally, we verified that miR-1249 suppressed CRC proliferation and angiogenesis by targeting VEGFA as well as inhibited CRC metastasis by targeting both VEGFA and HMGA2. Further studying showed that miR-1249 suppressed CRC cell proliferation, migration, invasion, and angiogenesis via VEGFA-mediated Akt/mTOR pathway as well as inhibited EMT process of CRC cells by targeting both VEGFA and HMGA2. Our study indicated that P53-induced miR-1249 may suppress CRC growth, metastasis and angiogenesis by targeting VEGFA and HMGA2, as well as regulate Akt/mTOR pathway and EMT process in the initiation and development of CRC. miR-1249 might be a novel the therapeutic candidate target in CRC treatment.

## Introduction

Colorectal cancer (CRC) is one of the most common malignancies worldwide, and tumor metastasis are the leading causes of morality in these patients^1^. Although new drugs including inhibitors of EGFR signaling and angiogenesis have elevated survival time in metastatic CRC patients^2^, metastatic CRC remains an incurable disease in most these patients. Therefore, a better understanding of the molecular mechanisms involving in initiation and development of CRC become urgent.

MicroRNAs (miRNAs) are a group of non-coding RNAs (18–22 nucleotide) that have been reported to be act as a tumor suppressor or oncogene via regulating their target gene through mRNA degradation, posttranscriptional repression or promoter activation^3–5^. Recently, mounting miRNAs have been shown to play key roles in multiple biological processes in CRC^6–8^, including cell tumor growth, metastasis, drug-resistance, angiogenesis and apoptosis, and have been identified as potential therapeutic and prognostic biomarkers in CRC diagnosis and treatment.

MiR-1249, located on 22q13.31, has been reported to be aberrantly expressed and closely associated with prognosis in hepatocellular carcinoma (HCC)^9^. Nevertheless, the function of miR-1249 and its underlying molecular mechanisms in CRC remain elusive. Tumor-suppressor P53 mutant or loss is regarded as a critical event in the development of tumor^10^. In recent years, increasing dysregulated miRNAs have been identified to be directly regulated by P53 and modulated their target genes which were crucial to tumor initiation, progression and metastasis^11,12^.

In this study, we found that miR-1249 was downregulated in CRC tissues and cell lines, and was closely correlated with pT stage, pN stage, TNM stage, and overall survival (OS). Moreover, we verified P53 could bind to the promoter of miR-1249 using luciferase reporter, and regulate the expression of miR-1249. Subsequently, enhanced the expression of miR-1249 resulted in a reduction of cell proliferation, metastasis and the ability of angiogenesis. Furthermore, we showed that miR-1249 inhibited CRC growth, metastasis, and angiogenesis by targeting vascular endothelial growth factor A (VEGFA) and high mobility group AT-hook 2 (HMGA2).

## Results

### miR-1249 was markedly downregulated in CRC cell lines and tissues

**Fig. 1:**
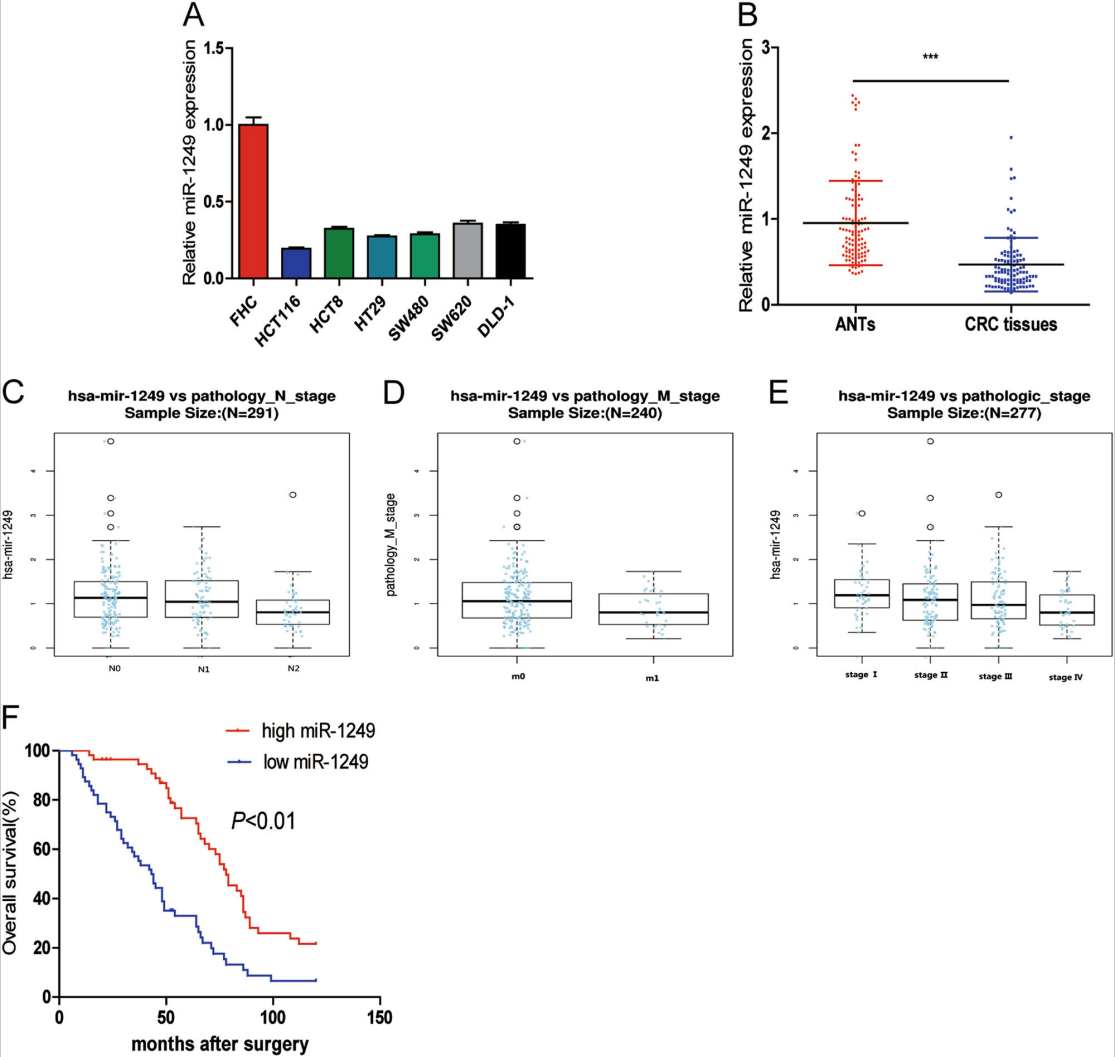
miR-1249 was downregulated in CRC cell lines and tissues. **a** Decreased miR-1249 expression was observed in all six CRC cell lines compared with the normal colonial epithelial cell (FHC). **b** qRT-PCR analysis of miR-1249 in 112 pairs of human CRC tissues and their adjacent normal tissues (ANTs). **c**–**e** miR-1249 was adverse correlated with pN stage (**c**), pM stage (**d**), and TNM stage (**e**). **f** Kaplan–Meier analysis of the correlation between miR-1249 expression levels and overall survival (OS) of 112 patients. ***P < 0.001

Firstly, we evaluated the expression of miR-1249 in six CRC cell lines (HCT116, HCT8, HT29, SW620, SW480, and DLD-1) and FHC. The results showed that miR-1249 was significantly downregulated in all of CRC cell lines compared with that in FHC (Fig. 1a).

Moreover, we examined the expression of miR-1249 in 112 pairs of human CRC tissues and matched adjacent normal tissues (ANTs). The results showed that miR-1249 was obviously downregulated in CRC tissues when compared to ANTs (Fig. 1b). These data indicated that miR-1249 might act a tumor suppressor in CRC.

To further explore the potential clinical significance of miR-1249 in CRC, firstly, linkedOmics (http://www.linkedomics.org/) was performed to detect whether miR-1249 expression were associated with clinicopathological parameters of CRC patients, and found that low levels of miR-1249 was markedly associated with high pN stage (N2) (Fig. 1c, *P* < 0.05), high pM stage(M1) (Fig. 1d, *P* < 0.05) and TNM stage (III/IV) (Fig. 1e, *P* < 0.05). Next, we measured miR-1249 expression by qRT-PCR in all these 112 patients and divided them into two groups (low and high miR-1249 expression), no significant association was found between miR-1249 and age, gender, tumor size, differentiation, CEA, and CA199. However, its expression was markedly correlated with pN stage, pM stage and TNM stage (Table 1). These results revealed that low miR-1249 expression was associated malignant progression in CRC patients. Additionally, Kaplan–Meier was performed to assess the prognostic value of miR-1249 in CRC, and found that patients in the high miR-1249 group achieved OS prolongation in comparison with those in the low miR-1249 group (Fig. 1f).

**Table 1:** Correlation between miR-1249 expression and different clinical characteristics

Characteristics	*n* = 112	miR-1249 expression		*P* value
		High (%) (*n* = 56)	Low (%) (*n* = 56)	
*Gender*				0.072
Male	74 (66.1%)	32 (57.1%)	42 (75.0%)	
Female	38 (33.9%)	24 (42.9%)	14 (25.0%)	
*Age (years)*				0.156
<60	36 (32.1%)	14 (25.0%)	22 (39.3%)	
≥60	76 (67.9%)	42 (75.0%)	34 (60.7%)	
*Tumor location*				0.130
Colon	59 (52.3%)	34 (60.7%)	25 (44.6%)	
Rectal	53 (47.7%)	22 (39.3%)	31 (55.4%)	
*TNM stage*				<0.001
I–II	65(58.0%)	43(76.8%)	22 (39.3%)	
III–IV	47(42.0%)	13(23.2%)	34 (60.7%)	
*Differentiation*				0.727
Low	27 (24.1%)	12 (21.4%)	15 (26.8%)	
Moderate	70 (62.5%)	37 (66.1%)	33 (58.9%)	
High	15 (13.4%)	7 (12.5%)	8 (14.3%)	
*pT stage*				0.237
T1–T2	13 (11.6%)	4 (7.1%)	9 (16.1%)	
T3–T4	99 (88.4%)	52 (92.9%)	47 (83.9%)	
pN stage				0.009
N0	66 (58.9%)	40 (71.4%)	26 (46.4%)	
N1	31 (27.7%)	11 (19.6%)	20 (35.7%)	
N2	15 (13.4%)	5 (9.0%)	10 (17.9%)	
pM stage				0.003
M0	96 (85.7%)	53 (94.6%)	43 (76.8%)	
M1	16 (14.3%)	3 (5.4%)	13 (23.2%)	
Tumor size				0.336
<5 cm	66 (58.9%)	30 (53.6%)	36 (64.3%)	
≥5 cm	46 (41.1%)	26 (46.4%)	20 (35.7%)	
CEA				0.554
<10 ng/ml	72 (64.3%)	34 (60.7%)	38 (67.9%)	
≥10 ng/ml	40 (35.7%)	22 (39.3%)	18 (32.1%)	
*CA199*				0.255
<10U/ml	51 (45.5%)	29 (41.1%)	22 (50.0%)	
≥10U/ml	61 (54.5%)	27 (58.9%)	34 (50.0%)	

Univariate and multivariate analysis were used to analyze miR-1249 expression and other clinicopathological parameters on the prognosis of CRC patients. Univariate analysis indicated that pM stage, TNM stage and miR-1249 expression were predictors for poor OS. Multivarivate analysis revealed that miR-1249 was an independent prognostic factor for OS in CRC patients(Table 2).

**Table 2:** univariate and multivariate analysis for OS in patients with CRC

Characteristics	Multivariate analysis for OS		Univariate analysis for OS	
	HR (95% CI)	*P*	HR (95% CI)	*P*
Gender (male/female)	–	–	1.158 (0.739–1.812)	0.522
Age (<60/≥60)	–	–	0.928 (0.618–1.551)	0.928
Tumor location (colon/rectal)	–	–	1.160 (0.754–1.785)	0.500
TNM stage (I–II/III–IV)	2.016 (1.152–3.526)	0.014	2.073 (1.339–3.209)	0.001
Differentiation (low/moderate/high)	–	–	1.322 (0.935–1.871)	0.114
PT stage (T1–T2/T3–T4)	–	–	0.674 (0.357–1.271)	0.223
PN stage (N0/N1/N2)	1.847 (1.162–2.936)	0.001	1.814 (1.182–2.785)	0.006
Distant metastasis(M0/M1)	3.170 (1.592–6.313)	0.001	2.702 (1.451–5.031)	0.002
Tumor size (<5 cm/≥5 cm)	–	–	0.654 (0.420–1.018)	0.060
CEA (<10 ng/ml/≥10 ng/ml)	–	–	0.775 (0.479–1.192)	0.228
CA199 (<10 U/ml/≥10 U/ml)	–	–	0.751 (0.488–1.156)	0.193
miR-1249 (low/high)	1.617 (1.092–2.671)	0.012	1.935 (1.211–3.013)	0.002

*CI* confidence interval, *HR* hazard ratio

### miR-1249 inhibited cell proliferation, migration, invasion and HUVECs tube formation

**Fig. 2:**
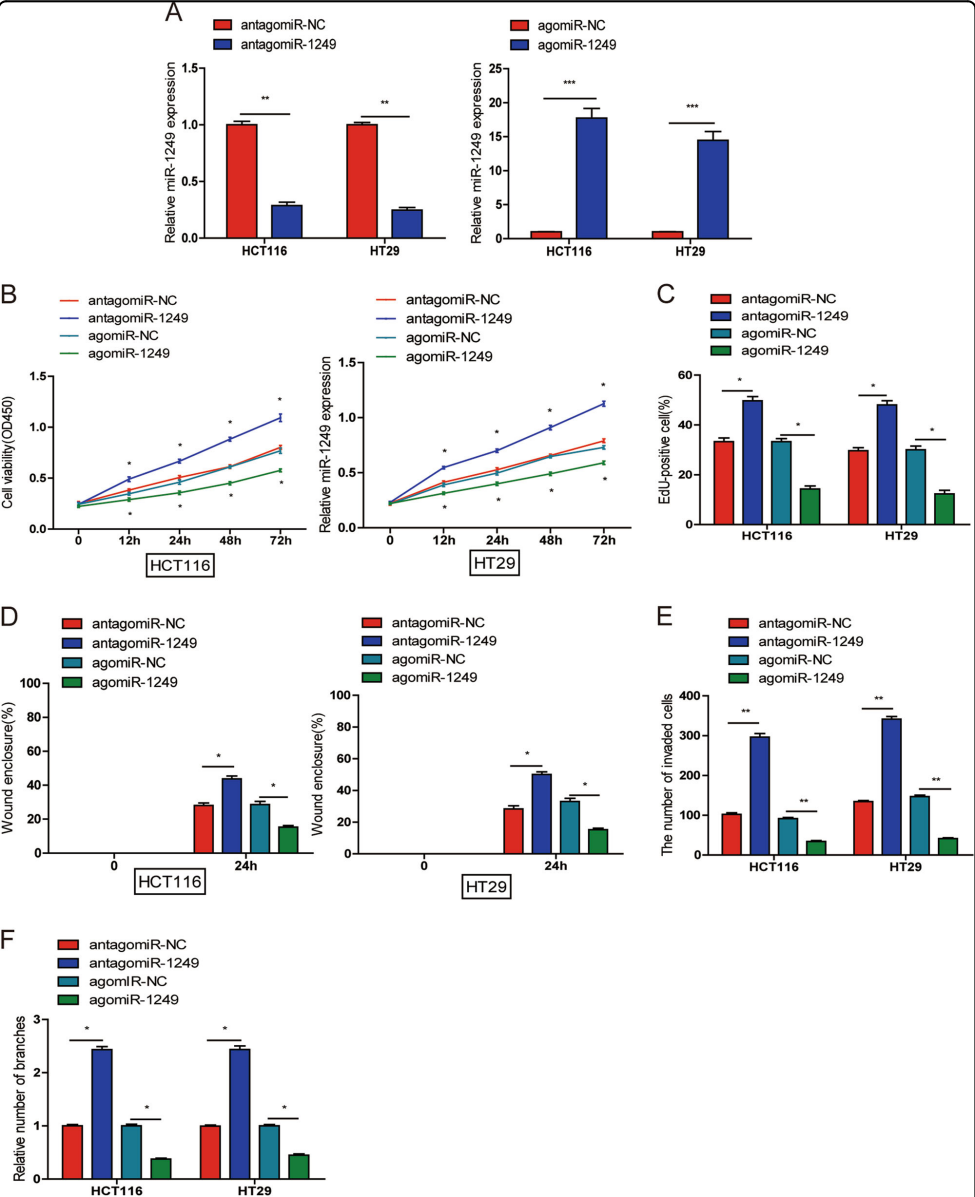
miR-1249 inhibited CRC cell proliferation, migration, invasion and HUVECs tube formation. **a** HCT116 and HT29 cells were transfected with antagomiR-1249, agomiR-1249 and corresponding negative control (antagomiR-NC, agomiR-NC). GAPDH was used as a internal control. **b**, **c** CCK-8 (**b**) and EdU (**c**) assays were used to evaluate the proliferation of transfected CRC cells(HCT116 and HT29). **d**, **e** wound-healing (**d**) and transwell assays (**e**) were performed to detect the ability of migration and invasion of transfected CRC cells. **f** HUVECs were cultured in TCM from CRC cells transfected with antagomiR-NC, antagomiR-1249, agomiR-NC and agomiR-1249. The number of tube branches were measured in 10 photographic fields randomly. Data were shown as mean ± SD of three independent experiments. *P < 0.05, ** *P* < 0.01, ***P < 0.001

We selected HCT116 and HT29 for agomiR-1249 and antagomiR-1249 transfection. As shown in Fig. 2a, the miR-1249 expression levels were obviously increased by agomiR-1249 and decreased by antagomiR-1249 in HCT116 and HT29 cells. MiR-1249 overexpression resulted in decreased cell proliferation, whereas inhibition of miR-1249 significantly increased cell proliferation when compared to their negative control, respectively (Fig. 2b, c and S1A). The migratory (Fig. 2d and S1B) and invasive (Fig. 2e and S1C) ability were increased in HCT116 and HT29 cells treated with antagomiR-1249, while both capabilities were reduced by agomiR-1249 in HCT116 and HT29 cells. Tumor conditioned medium(TCM) from CRC cells transfected with agomiR-1249 obviously inhibited tube formation of HUVECs, while TCM from CRC cells transfected with antagomiR-1249 markedly promoted tube formation of HUVECs (Fig. 2f and S1D). Based on above findings, we could conclude that miR-1249 suppressed CRC proliferation, migration, invasion, and HUVECs tube formation in vitro.

### P53 transcriptionally trans-activated miR-1249

**Fig. 3:**
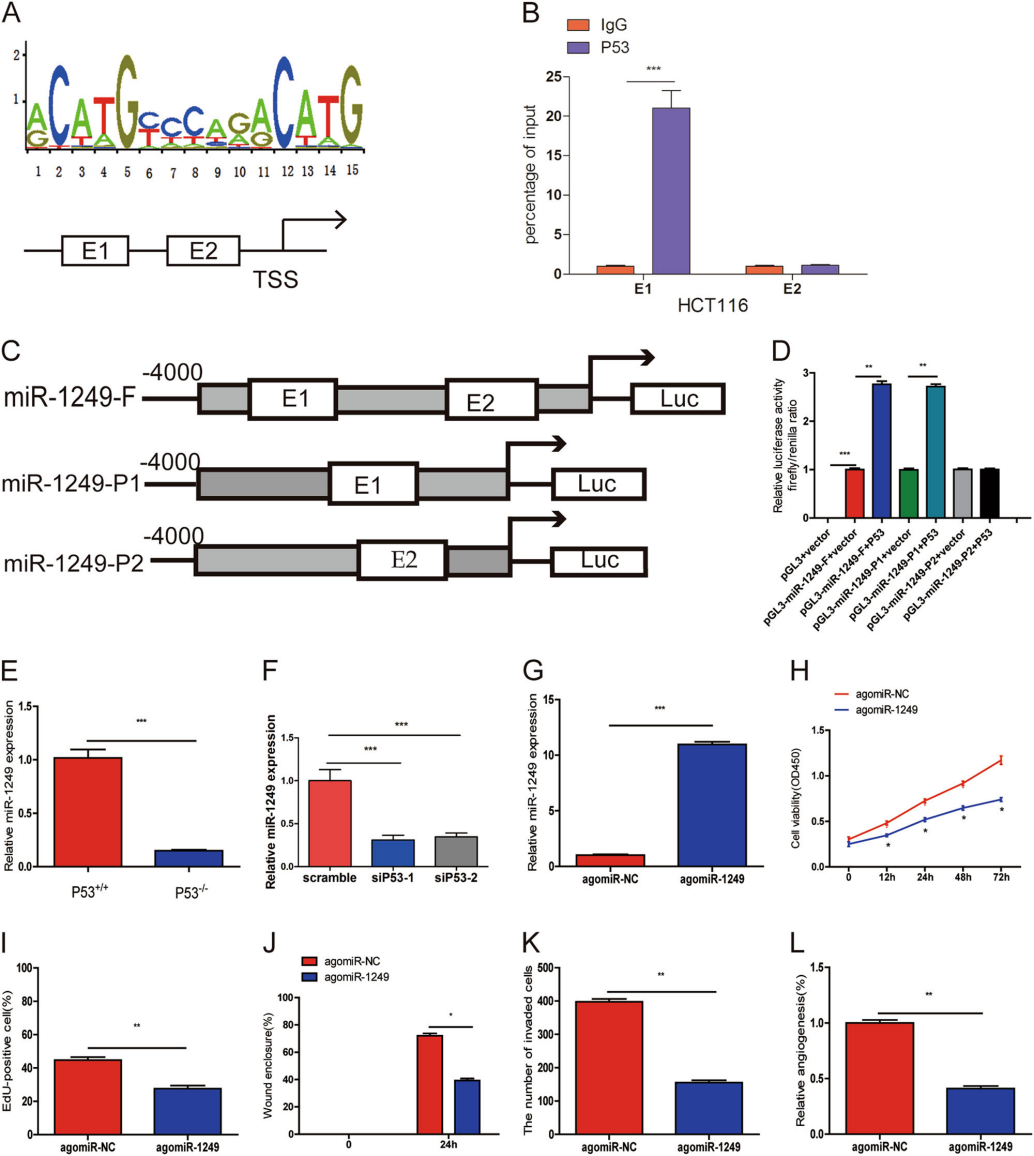
P53 induced miR-1249 expression as a transcription factor. **a** The predicted positions of puative P53 binding motif in −4000 bp human miR-1249 promoter. **b** Quantitative ChIP assays were performed to show direct binding of P53 to endogenous miR-1249 promoter regions. The primers designed for ChIP were provided in supplementary materials and methods. **c**, **d** A dual-luciferase reporter assay was used by cotransfecting with full length miR-1249 promoter(miR-1249-F) or deleted miR-1249 E1 or E2 fragment (miR-1249-P2 and miR-1249-P1) with P53 expression plasmid or blank vector in 293T cells. Results are presented as mean ± SD. **e** The expression of miR-1249 in P53^-/-^ and P53^+/+^ HCT116 cells. **f** The expression of miR-1249 was decreased after P53 knockdown in P53^+/+^ HCT116 cells. **g** P53^-/-^ HCT116 cells were transfected with agomiR-1249 or agomiR-NC. **h**–**l**. The ability of proliferation (**h**, **i**), migration (**j**), and invasion (**k**) was markedly increased in P53^-/-^HCT116 cells after after agomiR-1249 transfection. **l** HUVECs were cultured in TCM from P53^-/-^ HCT116 cells transfecting with agomiR-1249 or agomiR-NC. Data were shown as mean ± SD. All the experiments were performed more than three times. *P < 0.05, **P < 0.01, ***P < 0.001

To explore whether P53 could regulate miR-1249 at the transcriptional level, UCSC (http://genome.ucsc.edu/) was used to identified the miR-1249 promoter and Transfac and Jaspar database were employed to check for the potential P53 binding sites up to 4 Kb upstream of the transcriptional start site (TSS). A P53 binding site was identified at E1 (−3800/−3786 bp, gcctgcactgacttg) and at E2 (−2704/−2690 bp, ccatgcacaagcagg) upstream of the miR-1249 promoter TSS (Fig. 3a). To determine whether P53 could bind to the promoter of miR-1249, firstly, we designed two paired primers and ChIP assay was employed to detect which region in the miR-1249 promoter mediated P53-binding to the miR-1249 promoter. The ChIP data showed that P53 could bind to E1 sites (Fig. 3b). To further verified this results, we cloned the full promoter region of miR-1249 and E1 or E2 promoter region mutant region into the pGL3-basic vector (Fig. 3c). Our results showed that P53 induced the promoter activity of miR-1249 in 293T cells transfected with full promoter region of miR-1249. After transfecting with P53 expression plasmid, the P2 mutant (not containing the E1 P53 binding site) caused an obvious reduction in promoter activity, but this reduction was not observed in the P1 mutant (not containing the E2 P53 binding site) in comparison with the full-length promoter construct (Fig. 3d). These results showed that P53 could directly bind to E1 instead of the E2 on the miR-1249 promoter.

### P53-induced miR-1249 inhibited CRC growth, metastasis, and angiogenesis in vitro

Firstly, we examined the expression of miR-1249 in P53^+/+^ HCT116 and P53^−/−^ HCT116 cells, and found that miR-1249 was significantly downregulated in P53^−/−^ HCT116 cells in comparison with P53^+/+^ HCT116 cells (Fig. 3e). We also measured the expression of miR-1249 after P53 knockdown in P53^+/+^ HCT116 cells, and observed that miR-1249 expression levels were decreased after P53 knockdown in P53^+/+^ HCT116 cells (Fig. 3f). Next, we upregulated miR-1249 using agomiR-1249 in P53^−/−^ HCT116 cells, and qRT-PCR was confirmed the transfection efficiency (Fig. 3g). We detected that the proliferation (Fig. 3h, i and S2A), migration (Fig. 3j and S2B) and invasion (Fig. 3k and S2C) of P53^−/−^ HCT116 cells transfected with agomiR-1249 was markedly reduced when compared to that in P53^−/−^ HCT116 cells transfected with agomiR-NC. Moreover, TCM from P53^−/−^ HCT116 cells transfected with agomiR-1249 substantially inhibited HUVECs tube formation (Fig. 3l and S2D).

### P53-induced miR-1249 inhibited CRC growth, metastasis, and angiogenesis in vivo

**Fig. 4:**
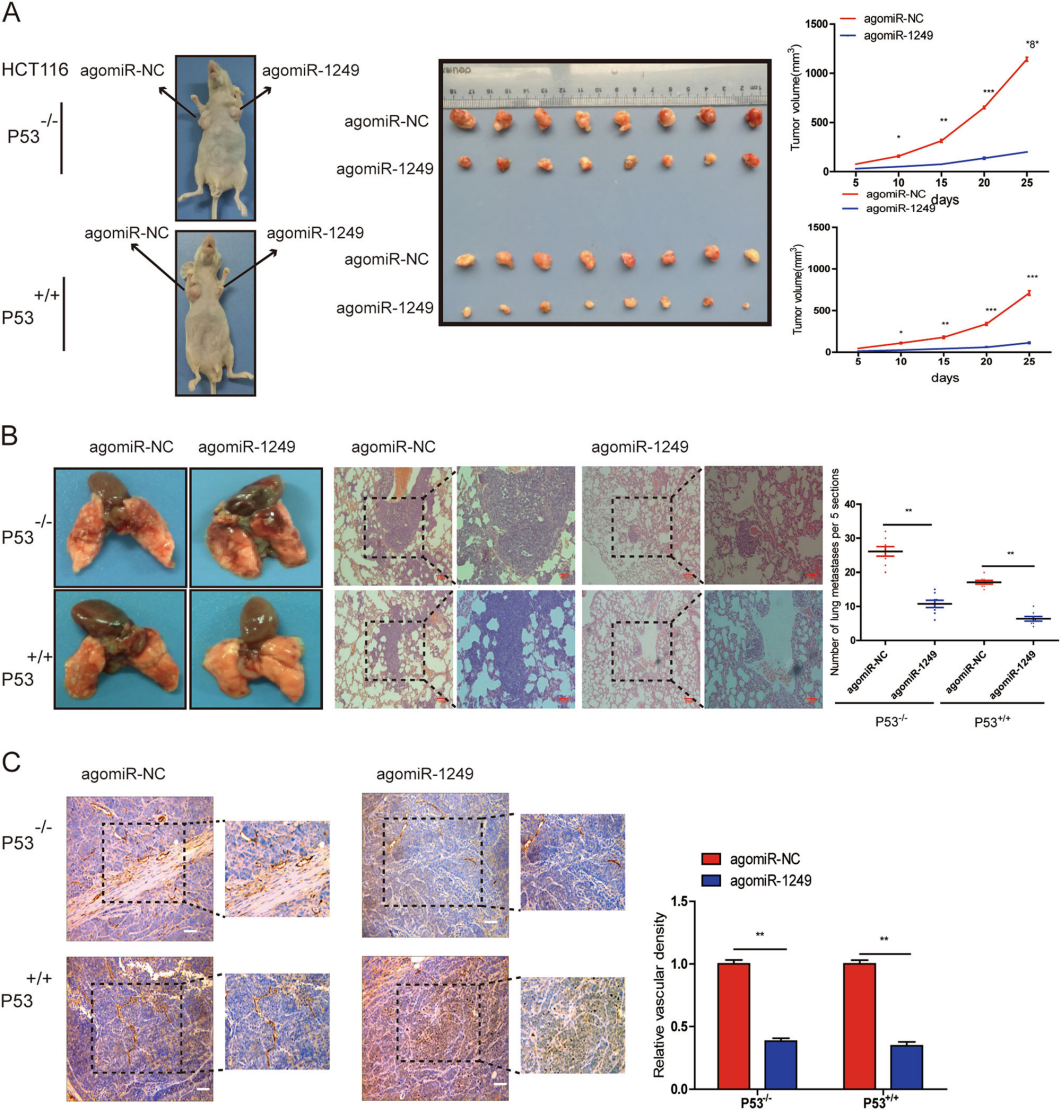
P53 induced miR-1249 inhibited tumor growth and distant metastasis in vivo. **a** The nude mice were injected with P53^-/-^ or P53^+/+^ HCT116 cells transfected with agomiR-1249 or agomiR-NC. The diameter of tumors were measured every 5 days. **b** Representative lungs and representative HE of lungs from mice. AgomiR-1249 group developed lower and smaller lung metastatic foci than agomiR-NC group. **c** CD31 expression were analyzed in xenografts tissues by IHC. Data were shown as mean ± SD. *P < 0.05, ** *P* < 0.01, ***P < 0.001

Based on our findings on miR-1249 in vitro, we further investigate the role of miR-1249 on tumor growth, metastasis and angiogenesis in vivo. An obvious reduction of tumor volume was observed in the miR-1249 overexpression cell lines(both P53^+/+^ and P53^−/−^ HCT116) in comparison with negative control (Fig. 4a).

Next, we analyzed the distant metastasis in the lung tissues from mice, and observed an obvious reduced number of metastatic nodules in the lungs of mice injected with agomiR-1249 compared with negative control (agomiR-NC) (Fig. 4b).

IHC of all xenograft tissues for CD31 (a marker for microvessel denoting increased angiogenesis) was performed to evaluate whether P53-induced miR-1249 had a role in CRC angiogenesis. We found a significant decrease in CD31 expression in tumors injected with agomiR-1249 compared with tumors injected with agomiR-NC (Fig. 4c).

Furthermore, we also found that miR-1249 knockdown significantly promoted HCT116 (P53^+/+^) cells growth and metastasis in vivo experiments (Fig S3A, S3B), and a markedly elevate CD31 expression in tumors injected with antagomiR-1249 compared with tumors injected with antagomiR-NC (Fig S3C).

### VEGFA and HMGA2 are both direct target genes of miR-1249

**Fig. 5:**
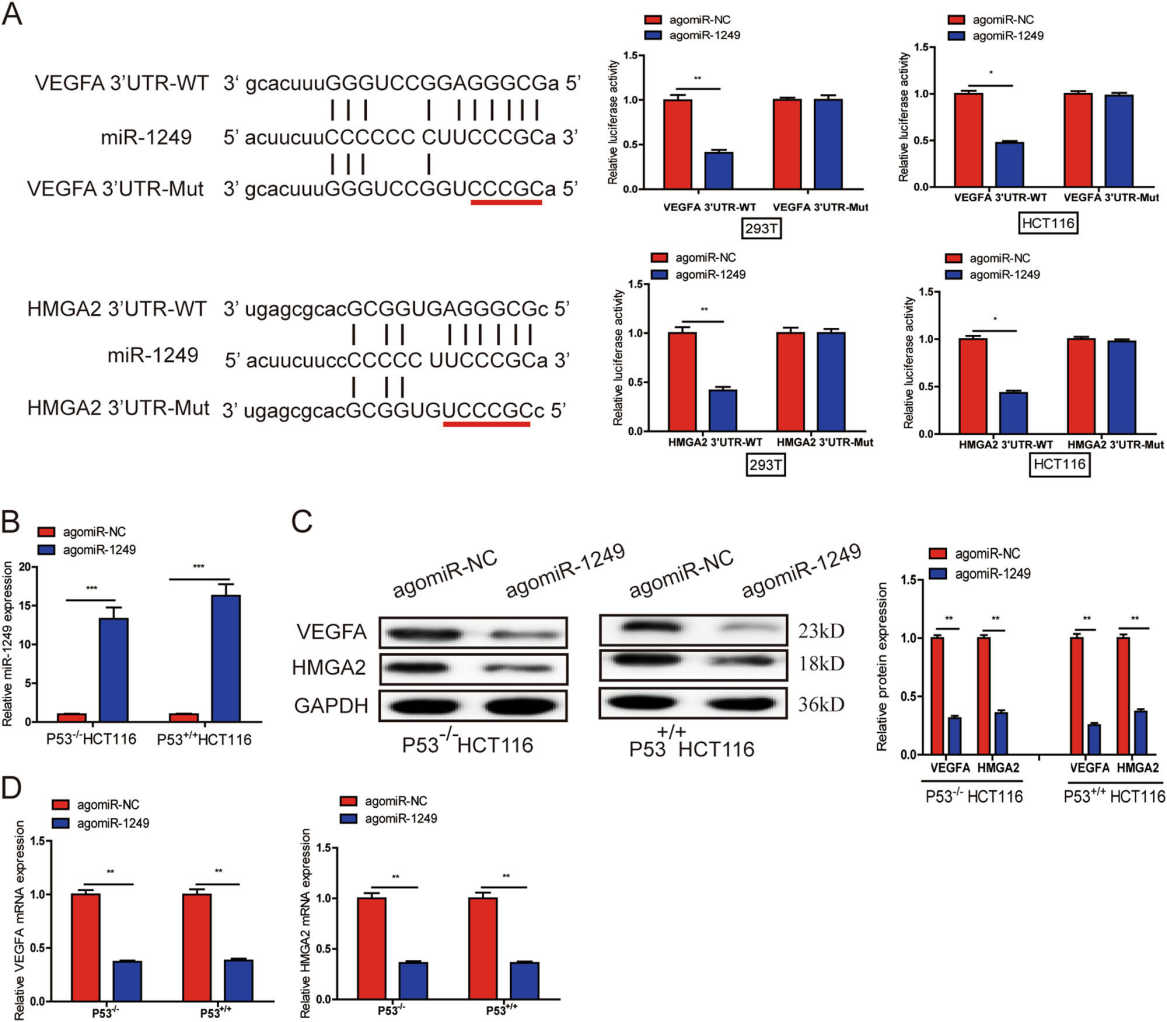
VEGFA and HMGA2 are both direct target genes of miR-1249. **a** miR-1249 and its putative binding sequence in the wild-type (WT) and mutant (Mut) 3’-UTR of VEGFA or HMGA2. Overexpression of miR-1249 obviously decreased the luciferase activity that carried WT but not Mut 3′-UTR of VEGFA and HMGA2 in 293T and HCT116 cells. **b**, **c** Upregulation of miR-1249 could significantly decreased the expression of VEGFA and HMGA2 both on protein (**b**) and mRNA (**c**) levels in P53^-/-^ and P53^+/+^ HCT116 cells. **d** Both VEGFA and HMGA2 mRNA were downregulated in xenografts tissues with high miR-1249. Data were presented as mean ± SD of three independent experiments. *P < 0.05, **P < 0.01, ***P < 0.001

To uncover the molecular mechanism of miR-1249, we used miRanda(www. microrna.org) and targetscan(www. targetscan.org) to identify the molecular targets of miR-1249. Of these genes, VEGFA and HMGA2 were selected to further study, since VEGFA and HMGA2 are both reported to be closely associated with CRC progression^13–15^. Next, a direct interaction of miR-1249 and VEGFA or HMGA2 was further substantiated via cloning the predicted wild-type (WT) and mutanted (Mut) 3′-UTR-binding site in a dual-luciferase reporter plasmid (Fig. 5a). Decreased luciferase activity after agomiR-1249 cotransfection in 293T and HCT116 cells was observed in the VEGFA and HMGA2 WT sequences, but the mutanted binding site didn’t change the luciferase activity (Fig. 5a). To explore whether these two genes were influenced by miR-1249, we transiently transfected P53^−/−^ and P53^+/+^ HCT116 cells with agomiR-1249 or agomiR-NC. Both these two genes on protein and mRNA levels were significantly downregulated after miR-1249 overexpression (Fig. 5b, c).

Moreover, we detected VEGFA mRNA and HMGA2 mRNA expression levels using qRT-PCR, and assessed the expression of VEGFA and HMGA2 protein using IHC and IF in xenograft tissues. We found both VEGFA and HMGA2 were downregulated in xenograft tissues with high miR-1249 when compared to xenograft tissues with low miR-1249 on mRNA and protein levels (Fig. 5d, S4A, S4B).

### VEGFA, HMGA2 could reverse the inhibitory effect caused by miR-1249 overexpression

**Fig. 6:**
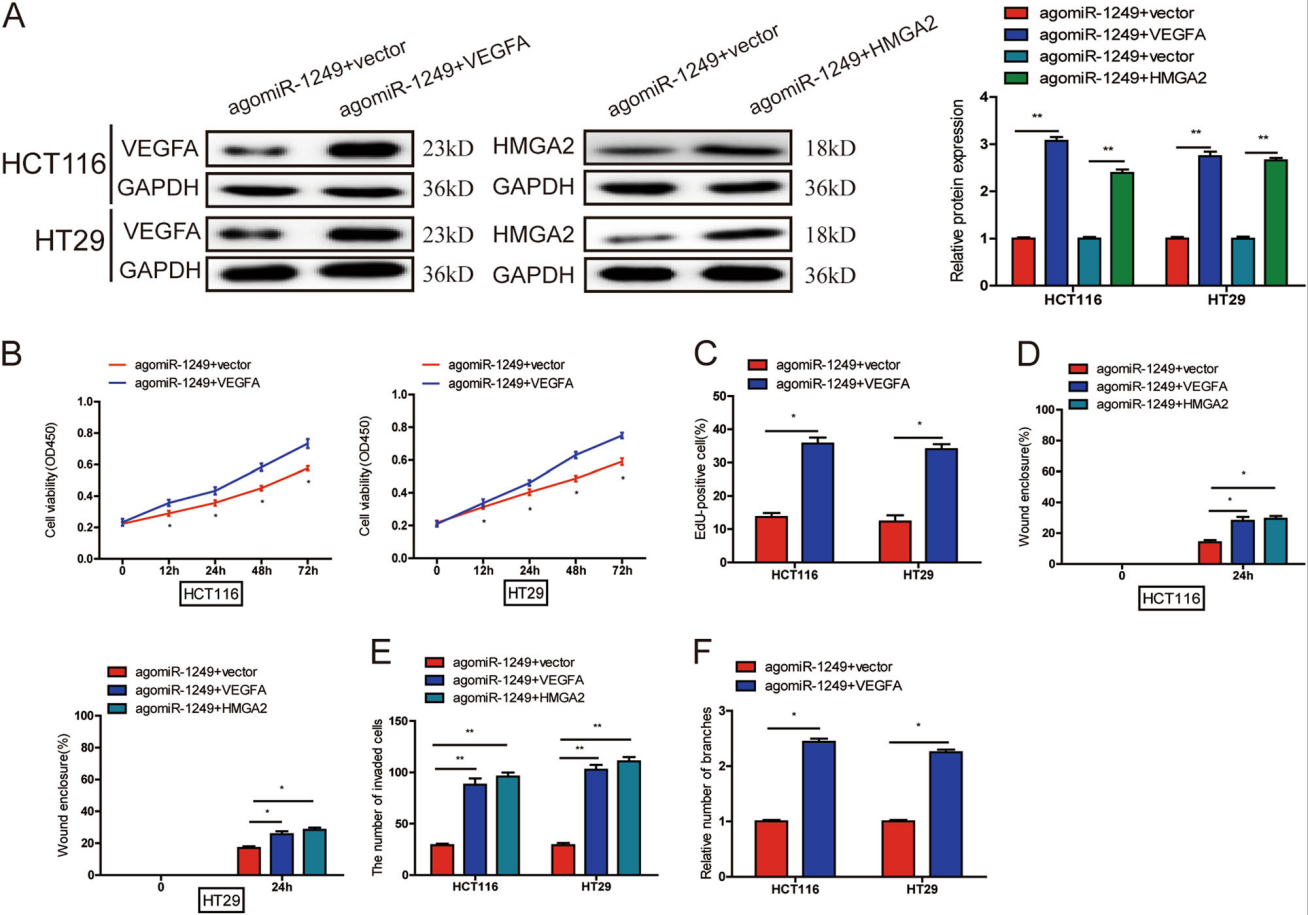
miR-1249 inhibited CRC growth and angiogenesis by VEGFA, and suppressed migration and invasion by both VEGFA and HMGA2. **a** miR-1249-overexpressioning HCT116 and HT29 cells that transfected with blank vector or pcDNA3.1-VEGFA and pcDNA3.1-HMGA2 were subjected to western blot for VEGFA and HMGA2. **b**, **c**. VEGFA upregulation could promote proliferation of miR-1249-overexpressing CRC cells using CCK-8 (**b**) and EdU (**c**). **d**, **e** VEGFA or HMGA2 upregulation could abrogate the effects of miR-1249-overexpressing on migration (**d**) and invasion (**e**) of CRC cells. **f** The ability of HUVECs cultured in TCM from VEGFA-upregulating CRC cells was obviously increased. Results were presented as mean ± SD. All the experiments were performed more than three times. *P < 0.05, **P < 0.01

To test whether miR-1249 exerted its effect on CRC progression by influencing VEGFA and HMGA2. miR-1249 over-expressing CRC cells were transfected with VEGFA or HMGA2 expression plasmid or blank vector (Fig. 6a). We found that upregulation of VEGFA resulted in a significantly increase of cell proliferation, migration, invasion, and HUVECs tube formation, while HMGA2 overexpression could substantially counteract the effects of upregulation of miR-1249 on the migratory and invasive ability of CRC cells (Fig. 6b, c and S5A, 6D and S5B, 6E and S5C, 6F and S5D). Collectively, miR-1249 inhibited CRC cell proliferation and HUVECs tube formation angiogenesis by targeting VEGFA, while suppressed CRC cell migration and invasion via both suppressing VEGFA and HMGA2.

### Clinical relevance of miR-1249, VEGFA, and HMGA2 expression in CRC patients

**Fig. 7:**
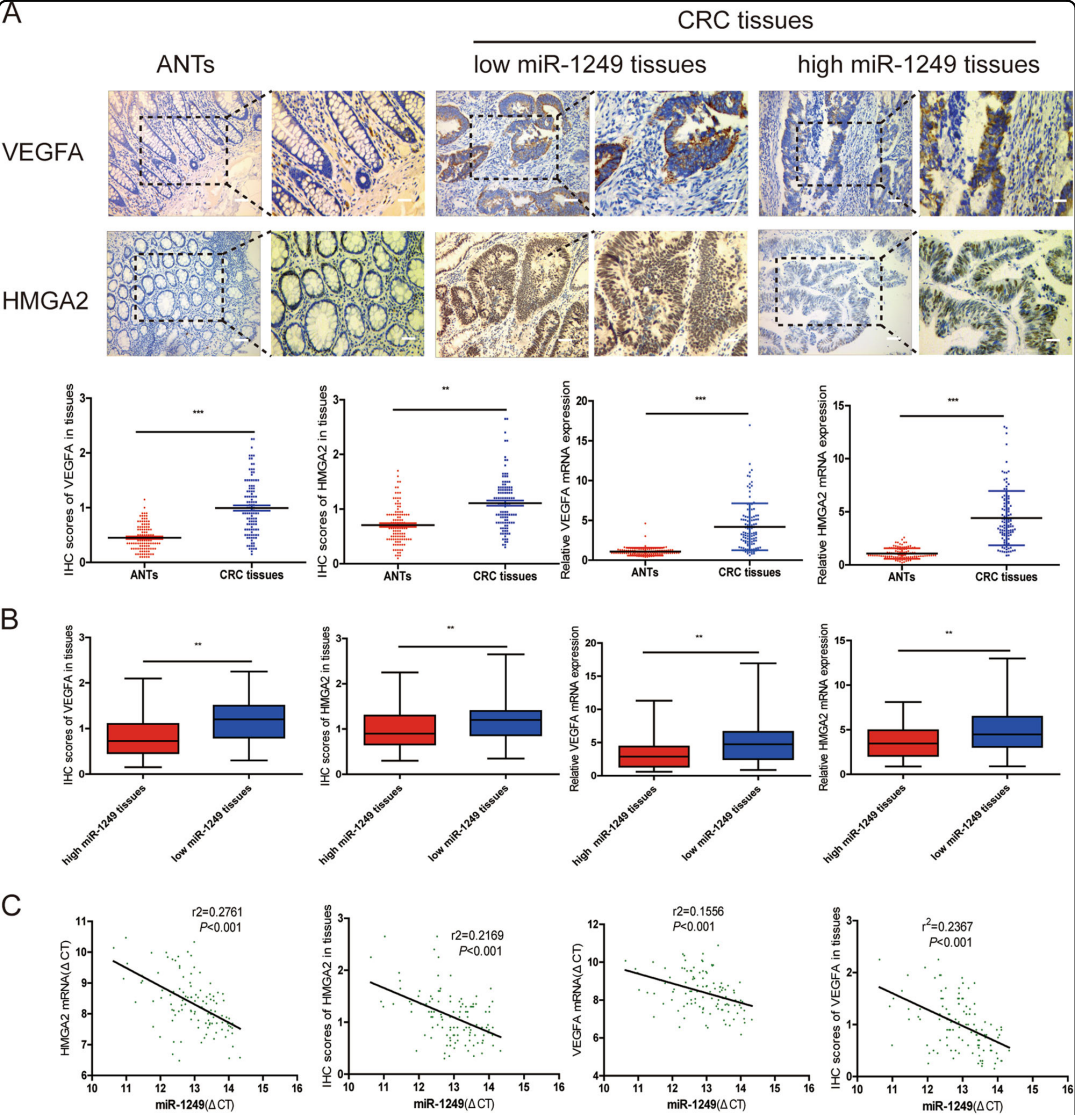
The expression of VEGFA and HMGA2 were negative correlated with miR-1249 in CRC tissues. **a** The statistical graph showed that IHC scores of VEGFA and HMGA2 in CRC tissues were significantly higher than those in matched adjacent normal tissues (ANTs). qRT-PCR analysis showed that both VEGFA and HMGA2 mRNA levels were higher than those in ANTs. **b** The statistical graph showed that IHC scores and mRNA of VEGFA and HMGA2 in low-miR-1249 CRC tissues were significantly higher than that of high-miR-1249 CRC tissues, respectively. **c** The relationship between miR-1249 and VEGFA and HMGA2 expression on protein and mRNA levels in CRC tissues. Data were shown as mean ± SD. **P < 0.01, ***P < 0.001

Given that miR-1249 downregulated VEGFA and HMGA2 in CRC cells to inhibit CRC progression, we next detected if this relationship existed in clinical tissue samples. We found that both VEGFA and HMGA2 on mRNA and protein levels in CRC tissues are overexpressed when compared to corresponding adjacent normal tissues (ANTs) (Fig. 7a). We also demonstrated that CRC tissues with high miR-1249 expression showed low VEGFA and HMGA2 mRNA as well as low IHC score of VEGFA and HMGA2. Reversely, CRC tissues with low miR-1249 expression tended to show high expression of VEGFA and HMGA2 both on mRNA and protein levels (Fig. 7b). Moreover, a reverse relationship was found between miR-1249 and VEGFA or HMGA2 (Fig. 7c). These results further verified that miR-1249 inhibited CRC progression by targeting both VEGFA and HMGA2.

### miR-1249 inhibited EMT process and repressed CRC progression via inactivating Akt/mTOR signaing pathway

**Fig. 8:**
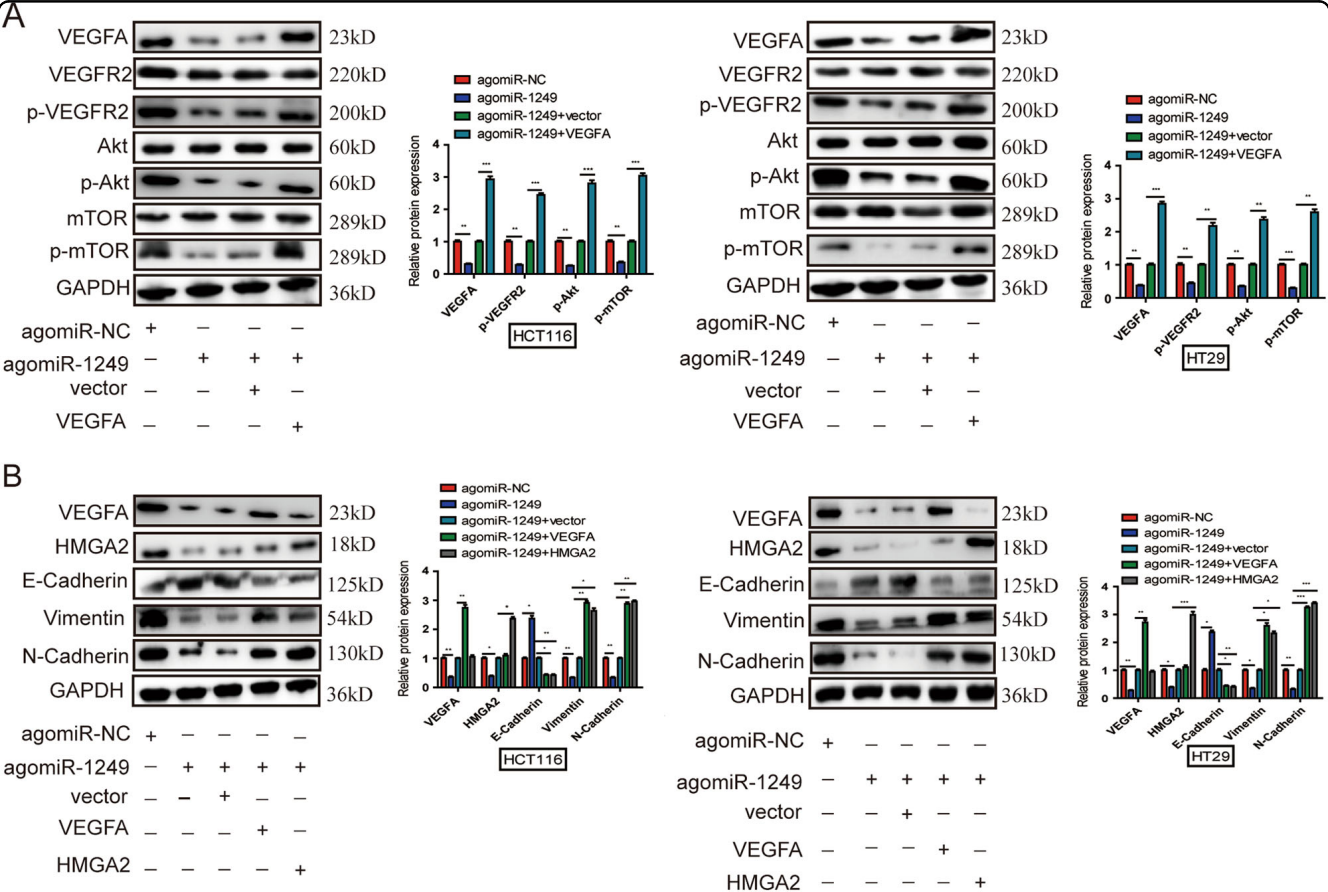
miR-1249 downregulated VEGFA and HMGA2 expression, and inhibited Akt/mTOR signaling pathway and EMT process. **a** The downregulation of VEGFA, p-VEGFR2, p-Akt and p-mTOR were countacted by VEGFA. **b** The downregulation of Vimentin and N-Cadherin and the upregulation fo E-Cadherin were countacted by both VEGFA and HMGA2. GAPDH was performed as a loading control. Data indicate mean ± SD of three independent experiments, *P < 0.05, **P < 0.01, *P* < 0.001

Previous studies have been reported that VEGFA could bind to vascular endothelial growth factor receptor 2(VEGFR2), and then activate PI3K and its downstream target Akt and mTOR which play crucial roles in tumor growth, metastasis, angiogenesis and induce the epithelial–mesenchymal transition (EMT)^16–18^. HMGA2 plays a key role in EMT and induces invasion and metastasis of human cancers^19^. To explore the molecular mechanism of miR-1249-induced cancer progression, here, we noted that overexpressing miR-1249 decreased the expression of VEGFA, phosphorylated VEGFR2 (p-VEGFR2), phosphorylated Akt (p-Akt) and phosphorylated mTOR (p-mTOR), and upregulation of VEGFA could countact the downregulation of p-VEGFR2, p-Akt and p-mTOR in HCT116 and HT29 cells (Fig. 8a). Decreased expression of N-cadherin, Vimentin and increased E-cadherin expression levels after miR-1249 overexpression were found, while both VEGFA and HMGA2 overexpression could increase the protein expression of Vimentin and N-cadherin, and decrease the E-cadherin protein levels in HCT116 and HT29 cells (Fig. 8b). Based on above findings, we could conclude that miR-1249 suppressed CRC progression via stabilising the epithelial phenotype and inactivating Akt/mTOR signaling pathway.

## Discussion

Increasing miRNAs have been reported to be involved in the biological process of CRC, and aberrant expression of miRNAs is closely associated with tumor growth, metastasis and angiogenesis in CRC^20,21^. Therefore, a better understanding of miRNAs concerning gene networks may bring new insights into diagnosis and treatment of malignancies.

In this study, low miR-1249 levels were detected in the majority of CRC tissues compared with those in ANTs. Moreover, miR-1249 expression was obviously adverse correlated with pN, pM, TNM stage, and OS. Consequently, we found miR-1249 to have considerable tumor growth, metastasis, and angiogenesis suppressor effects and demonstrated that miR-1249 suppressed CRC cell proliferation, migration, invasion, and HUVECs tube formation in vitro.

MiRNAs have been recently reported to be as key components of the signaling cascades which regulate tumor suppression exerted by a well-documented transcription factor (TF) P53^22,23^. Chung Chang et al.^24^ reported that P53 regulated EMT and stem cell properties via modulating miR-200c. Taewan Kim et al.^25^ reported that miR-200 family which target ZEB1 and ZEB2 mRNAs were regulated by P53 at the transcriptional level. Here, we found miR-1249 is a direct target of P53, which was verified by luciferase reporter assay. We examined the expression of miR-1249 in P53^+/+^ and P53^-/-^ HCT116 cells, and found that miR-1249 was markedly decreased in P53^-/-^HCT116 cells as compared to that in P53^-/-^ HCT116 cells. Moreover, the ability of CRC cell proliferation, metastasis and angiogenesis was significantly decreased after transfecting with agomiR-1249 in P53^-/-^ HCT116 cells both in vitro and in vivo.

VEGFA and HMGA2 have both been reported to be dysregulated and promoted tumor progression in various cancers^26,27^. Firstly, we proved that VEGFA and HMGA2 were both direct target genes of miR-1249 using dual luciferase reporter assays. Furthermore, we found that the expression of VEGFA and HMGA2 on mRNA and protein levels were both downregulated after miR-1249 overexpression in CRC cells and xenograft tissues.

It was well accepted that miRNAs exert their functions through their target genes, Alip Ghosh et al. have reported that miR-199a-3p suppressed tumor growth, metastasis and angiogenesis by targeting VEGFA, VEGFR1, VEGFR2, HGF, and MMP2 in HCC^28^. Multiple miRNAs have been reported to suppress tumor growth, metastasis and angiogenesis via inhibiting the expression of their target gene VEGFA^29,30^. Here, we found miR-1249 suppressed CRC cell growth, metastasis and HUVECs tube formation by inhibiting VEGFA expression. and we here observed that miR-1249 suppressed CRC cell migration and invasion by targeting inhibiting HMGA2 expression.

Additionally, we also investigated the correlation between the expression of miR-1249 and VEGFA or HMGA2 in CRC tissues, our results showed that both VEGFA and HMGA2 were negative correlate with miR-1249 in CRC tissues.

Accumulating evidences have showed that miRNAs were closely associated with EMT in CRC, O’Brien et al. showed that miR-200 family played pivotal role in EMT in colorectal carcinogenesis^31^. In the present study, we found that miR-1249 could upregulate the expression of E-Cadherin and downregulate the expression of Vimentin and N-Cadherin, while the increased E-Cadherin and decreased Vimentin and N-Cadherin were partially abrogated after upregulation of VEGFA and HMGA2.

These data together revealed that both VEGFA and HMGA2 were key targets of miR-1249 to inhibit EMT.

Several studies have shown that Akt/mTOR signaling pathway played a key role in varieties of biological processes of cancers, such as proliferation, metastasis and angiogenesis, for example, miR-92b-3p/Gabra3 axis has been reported to modify pancreatic cancer cell growth and metastasis via regulating Akt/mTOR signaling pathway^32^. Here, we observed that miR-1249 suppressed CRC cell proliferation, metastasis and angiogenesis partially through VEGFA-mediated Akt/mTOR signaling pathway.

In conclusion, our study found that miR-1249 was downregulated in CRC tissues and cell lines, and was negative correlate with pN stage, pM stage, TNM stage and overall survival. Moreover, we showed that P53-induced miR-1249 inhibited CRC growth, metastasis and angiogenesis in vitro and vivo. Additionally, functional studies showed that miR-1249 suppressed CRC cell proliferation and angiogenesis by targeting VEGFA, while inhibited CRC cell migration and invasion by both VEGFA and HMGA2. An inverse expression pattern was also observed between miR-1249 and VEGFA or HMGA2 in CRC tissues. Furthermore, we found that miR-1249 inhibited EMT process by targeting VEGFA and HMGA2 as well as suppressed CRC initiation and development via VEGFA-mediated Akt/mTOR signaling pathway. Our findings supported the idea that miR-1249 played a crucial role in CRC progression and verified that miR-1249 was a potential effective target of treating CRC.

## Materials and methods

### Patients and tissues specimens

One hundred and twelve CRC tissues and matched ANTs were collected from Nanjing First Hospital, Nanjing Medical University between January 2001 and December 2007. None patients achieve chemotherapy or radiotherapy prior to surgery. All these samples were immediately snap frozen in liquid nitrogen and stored at −80 °C until RNA extraction. Informed consent were obtained from all these patients and this study was approved by the Institutional Review Board of Nanjing First Hospital, Nanjing Medical University. Clinicopathological characteristic of these patients are listed in Table 1.

### Cell culture and transfection

Human CRC cell lines HCT116 (P53^+/+^), HT29, SW480, SW620, HCT8, DLD-1, a normal colonial epithelial cell (FHC) and human umbilical vein endothelial cells (HUVECs) were obtained from the American Type Culture Collection (ATCC; USA). The P53^-/-^ HCT116 cells were previously obtained from the Johns Hopkins University Genetic Resources Core Facility. The cells were cultured in Dulbecco’s Modified Eagle Medium (DMEM, Gibco, USA)with 100U/ml penicillin, 0.1 mg/ml streptomycin and 10% fetal bovine serum (FBS, Gibco, USA) at 37 °C supplied with 5% CO_2_ atmosphere. AgomiR-1249, antagomiR-1249, small interferece RNA (siP53-1, siP53-2) targeting P53, pcDNA3.1-VEGFA, pcDNA3.1-HMGA2, pcDNA3.1-P53 and their corresponding negative control (agomiR-NC, antagomiR-NC, siP53 and blank vector) were transfected into cells using Lipofectamine^TM^ 2000 (Invitrogen, USA) in accordance with the manufacturer’s protocol.

### Cell counting kit-8 (CCK-8)

CCK-8 (KeyGEN BioTECH, China) was performed to evaluate cell proliferation according to the manufacturer’s instructions. Approximately 1 × 10^3^ transfected cells were seeded into a 96-well plate. After 12, 24, 48, and 72 h, 10 µl CCK-8 test solution were added into a 96-well plate, and incubated for 3 h. The absorbance at 450 nm was measured in a microplate reader(Infinite M200 Pro, Tecan).

### EdU assay

1.5 × 10^5^ transfected cells were seeded into a 24-well plate overnight. Next day, 50 µM 5-ethynyl-2′-deoxyuridine(EdU, Cell Light EdU DNA imaging Kit, RiboBio, China) were added and the cells were grown for additional 2 h. The cells were stained in accordance with following instructions: cells were fixed with 4% paraformaldehyde at room temperature for 30 min, then add glycine (2 mg/ml) for 5 min and add 0.5% Trion X-100 for 10 min. After washing for two times using PBS, the cells were stained with apollo^®^ fluorescent dye and Hoeschst 33342. The images(×200) were captured using inverted microscopy (Nikon, Japan) and the percentage of EdU-positive cells was detected from five random fields in three wells.

### Cell migration assays

The migration of transfected cells were evaluated by wound healing assay. 3 × 10^5^ cells/well were seeded into 6-well plates, after cells reached 90% confluence, a sterilized 200 µl pipette tip was performed to make a straight scratch in the wells. A inverted microscopy (Nikon, Japan) was used to capture the images.

### Cell invasion assays

The invasion of transfected cells was assessed using Transwell assay. In brief, 3 × 10^5^ cells suspended in 200 µl serum free DMEM were cultured into the upper chamber precoated with 100 µl 5% matrigel(BD Biosciences, USA), and 500 µl DMEM containing 15% FBS was added into the lower chamber. After 24 h, cells that didn’t invade were removed by a cotton swab. The invaded cells were fixed with 100% methanol for 15 min and stained with crystal violet for 10 min. A inverted microscopy (Nikon, Japan) was used to capture the images.

### HUVECs tube formation

HUVECs (10 × 10^5^) were suspended into the tumor conditioned medium (500 µl, TCM) with 10% FBS and planted into a 24-well plate precoated with matrigel (200 µl/well, BD Bioscience). Tube formation was detected and photographed using a inverted microscopy (Nikon, Japan) after 6 h of incubation at 37 °C.

### Chromatin immunoprecipiation assay

Chromatin immunoprecipiation (ChIP) assay was performed using EZ-Magna ChIP Kit (Millipore, Billerica, USA). Briefly, HCT116 cells were treated with 1% formaldehyde solution for 15 min at room temperature, and incubated with 125 nM glycine for 5 min. DNA fragments ranging 200–300 bp were generated using sonication. Antibodies including anti-P53 (10442-1-AP, Proteintech, China) and IgG were employed for each immunoprecipitation. qPCR was used to analyze the precipitated DNA.

### Luciferase reporter assay

Jaspar database((http://jaspar.genereg.net/) was employed to identify the P53-binding site in the promoter region of miR-1249. The different fragment sequences of miR-1249 promoter were synthesized and inserted into pGL3-basic vector, and then cotransfected with P53 expression plasmid into 293T cells. Wild type of VEGFA 3′-UTR, mutated VEGFA 3′-UTR, Wild type of HMGA2 3′UTR and mutated HMGA2 3′-UTR were contransfected with agomiR-1249 or negative control (agomiR-NC) using Lipofectamine^TM^ 2000 (Invitrogen, USA). Dual-luciferase reporter assay system (Promega, Madison, WI, USA) was employed to measure the relative luciferase activity and normalized to renilla luciferase activity.

### RNA extraction and quantitative real-time PCR

TRIzol Reagent (Invitrogen, USA) was used to extract total RNA following manufacturer’s instructions. MiR-1249 expression was measured by a Hairpin-it^TM^ microRNA and U6 snRNA normalization RT-PCR quantitation kit (Genepharma, China). For VEGFA mRNA and HMGA2 mRNA, complementary DNA (cDNA) was synthesized using PrimeScript^TM^ reagent kit with gDNA Eraser (Takara, Dalian, China) and qRT-PCR was analyzed using SYBR Premix Ex Taq kit (Takara, Dalian, China). The relative expression of miRNA or mRNA was analyzed using 2^-ΔΔCT^ method. All results were normalized to GAPDH or U6. The primers of VEGFA, HMGA2 mRNA, and GAPDH are listed in Supplementary table 1.

### Western blot

Total protein extracted from cells lysis buffer supplemented with protein inhibitor, phosphatase inhibitor and PMSF (KeyGEN BioCHEM, Nanjing, China.) and an equal amount of 30 µg protein was separated by 10% SDS-PAGE gel and transferred to polyvinylidence difluoride (PVDF) membranes (Millipore, USA). The membranes were blocked with 5% bovine serum albumin (BSA, USA) in TBS-tween, followed by incubating with primary antibodies: anti-VEGFA(ab52917, Abcam, UK), anti-HMGA2 (#8179, Cell Signaling Technogy, USA), anti-VEGFR2 (1:1000, ab39256, abcam, UK), anti-phospho(Y1214)-VEGFR2 (1:1000, ab5475, abcam, UK), ant-Akt (#9272, cell signaling technology, USA), anti-phospho(Ser473)-Akt (1:2000, #4060, cell signaling technology, USA), ant-mTOR (1:1000, #2983, Cell Signaling Technogy, USA), anti-phospho(Ser2448)-mTOR (1:1000, #5536, Cell Signaling Technogy, USA), rabbit anti-N-cadherin (1:1000, 22018-1-AP, proteintech, China), rabbit anti-E-cadherin (1:1000, 20874-1-AP, proteintech, China), rabbit anti-vimentin (1:1000,10366-1-AP, proteintech, China) and rabbit anti-GAPDH (1:10,000, ab9485, abcam, UK). Proteins were then detected by enhanced chemiluminescence system (ECL) reagent (KeyGEN BioTECH, China) after incubation with secondary antibodies for 1 h at room temperature.

### Immunohistochemistry

The CRC tissues, adjacent normal tissues and xenograft CRC tissues were collected, paraffin embedded and cut into 4µl-thick sections. Sections were incubated with rabbit anti-VEGFA, anti-HMGA2 and anti- CD31 at 4 °C overnight, followed by secondary antibodies. The sections were visualized under a inverted microscope(Nikon, Japan) at ×200 or ×400 magnification. The intensity of staining was scored by two independent pathologist in the following four categories: no staining = 0, weak staining = 1, moderate staining = 2, and strong staining = 3. The stain-positive were scored into four grades: 0 (0%), 1 (1–33%), 2 (34–66%), and 3(67–100%). The finally ICH score was calculated by multiplying the percentage of positive cells with the intensity score. The microvessel density (MVD) was assessed using CD31 IHC staining.

### Immunofluorescence staining

Tissue sections were blocked with 5% bovine serum albumin(BSA, sigma). Next, the sections were incubated at 4 °C overnight with primary antibodies, followed by incubation with Goat Anti-Rabbit IgG (H + L) Daylight 488(BS10017, Bioworld, China) for 1.5 h at room temperature. The nuclear of cells were stained with DAPI(sigma) for 10 min at room temperature and images (×200) were captured by fluorescence microscope (Nikon, Japan).

### In vivo experiments

All animal experiments were approved by the animal care Committee of Nanjing First Hospital, Nanjing Medial University(acceptance No. SYXK 20160006). For xenograft model, 1 × 10^7^ P53^+/+^ or P53^-/-^ HCT116 cells in 0.2 ml PBS were subcutaneously injected into the armpit region of 32 six-weeks old male BABL/c nude mice which were divided into four groups (*n* = 8 each group), after tumor formed (4 day after injection), 2 nmol agomiR-1249 and antagomiR-1249 or its negative control (agomiR-NC and antagomiR-1249) were injected into the tumors. The injections were used at an interval of 3 days (day 4, day 7, day 10, day 13, day 16, day 19, day 22), the tumors were measured every 5 days. The mice were sacrificed and the volume of tumors were measured after 3 days of the last injection. The volume of tumors was calculated using the following equation: *V* = (*L* × *W*^2^)/2, *L* is the length and *W* is the width of tumor.

For the metastasis experiments, 2 × 10^6^ P53^+/+^ and P53^-/-^ HCT116 cells in 0.1 ml PBS were injected into tail vein of mice, 100 nmol agomiR-1249 or agomiR-NC as well as antagomiR-1249 or antagomiR-1249 was injected into tail vein three times. The mice were sacrificed after 8 weeks of injection, and the lung tissues were isolated from these mice, and tissue sections were stained using hematoxylin and eosin (HE). An inverted microscope (Nikon, Japan) was used to counted the micrometastases in the lung tissues.

### Statistical analysis

All experiments were performed in triplicate. The data are expressed as mean ± SD, and analyzed by SPSS 22.0 software. The Student’s *t*-test, one-way analysis of variance (ANOVA) were performed to detected the differences between groups. Pearson’s Mann–Whitney *U*-test or *χ*^2^ test was used to analyze the relationship between expression of miR-1249 and clinicopathological features. Kaplan–Meier method was applied to assess OS. The survival curves were compared with log-rank test. Follow-up time was censored if the patient was lost to follow-up. Cox proportional hazards model was used to perform multivariate analysis and calculate the 95% confidence interval (95% CI). A statistically significant difference was considered as *P* < 0.05.

## Electronic supplementary material

supplementary Table 1

Supplementary Figure
