# Supplementary material for: P53-induced miR-1249 inhibits tumor growth, metastasis, and angiogenesis by targeting VEGFA and HMGA2
Source: Cell Death Dis. 2019 Feb 12;10(2):131. doi: 10.1038/s41419-018-1188-3 (PMC6372610; doi:10.1038/s41419-018-1188-3)
Supplement: Supplementary file 1 — supplementary Table 1 [file 41419_2018_1188_MOESM1_ESM.docx]

**The primers of HMGA2, VEGFA and GAPDH mRNA**

| HMGA2 | Forward: 5′-CATCAGCCCAGGGACAAC-3′ |
| --- | --- |
|  | Reverse: 5′-GAGAGGGCTCACAGGTTGG-3′ |
| VEGFA | Forward: 5’-TGGCTCACTGGCTTGCTCTA-3’ |
|  | Forward: 5’-ATCCAACTGCACCGTCACAG-3’ |
| GAPDH | Forward: 5'-CGAGATCCCTCCAAAATCAA-3' |
|  | Forward: 5'-TGTGGTCATGAGTCCTTCCA-3' |

**The primers of qChIP**

| E1 region | Forward: GGAGGACGCCTCATAAACTCTTG |
| --- | --- |
|  | Reverse: AAACAAAGGCTTGGCCAGATAA |
| E2 region | Forward: CAAGAAGGTGTATGACAACCGGG |
|  | Reverse: AATGACGTTAAGAGGACCAGGGA |
