# Supplementary material for: P53-induced miR-1249 inhibits tumor growth, metastasis, and angiogenesis by targeting VEGFA and HMGA2
Source: Cell Death Dis. 2019 Feb 12;10(2):131. doi: 10.1038/s41419-018-1188-3 (PMC6372610; doi:10.1038/s41419-018-1188-3)
Supplement: Supplementary file 2 — Supplementary Figure [file 41419_2018_1188_MOESM2_ESM.docx]

**Fig S1.** A-D. Representive amages of EdU(A,×200 ), wound healing(B, ×100), transwell(C, ×200) and tube formation(D, ×100) in antagomiR-NC group, antagomiR-1249 group, agomiR-NC group and agomiR-1249 group.

**Fig S2.** Representive amages of EdU(A,×200 ), wound healing(B, ×100), transwell(C, ×200) and tube formation(D, ×100) in P53^-/-^HCT116 cells transfected with agomiR-1249 or agomiR-NC.

**Fig S3.** miR-1249 knockdown promoted CRC cells growth, metastasis and angiogenesis. (A). The nude mice were injected with P53^+/+^ HCT116 cells transfected with antagomiR-1249 or antagomiR-NC. The diameter of tumors were measured every 5 days. (B). Representative lungs and representative HE of lungs from mice. AntagomiR-NC group developed lower and smaller lung metastatic foci than antagomiR-1249 group. C. CD31 expression were analyzed in xenografts tissues from antagomiR-NC group and antagomiR-1249 group by IHC. **P*<0.05, ** *P*<0.01, ****P*<0.001.

**Fig S4.** Representive amages of EdU(A,×200 ), wound healing(B, ×100), transwell(C, ×200) and tube formation(D) in HCT116 and HT29 cells cotransfected with agomiR-1249 and VEGFA or blank vector.

**Fig S5.** VEGFA and HMGA2 was downregulated in xenograft tissues with high miR-1249 compared with these in xenograft tissues with low miR-1249. A, B. IHC(A) and IF(B) staining of VEGFA and HMGA2 expression in subcutaneous tumors from nude mice injected P53^-/-^ /agomiR-NC or agomiR-1249 or P53^+/+^/agomiR-NC or agomiR-1249 HCT116 cells.


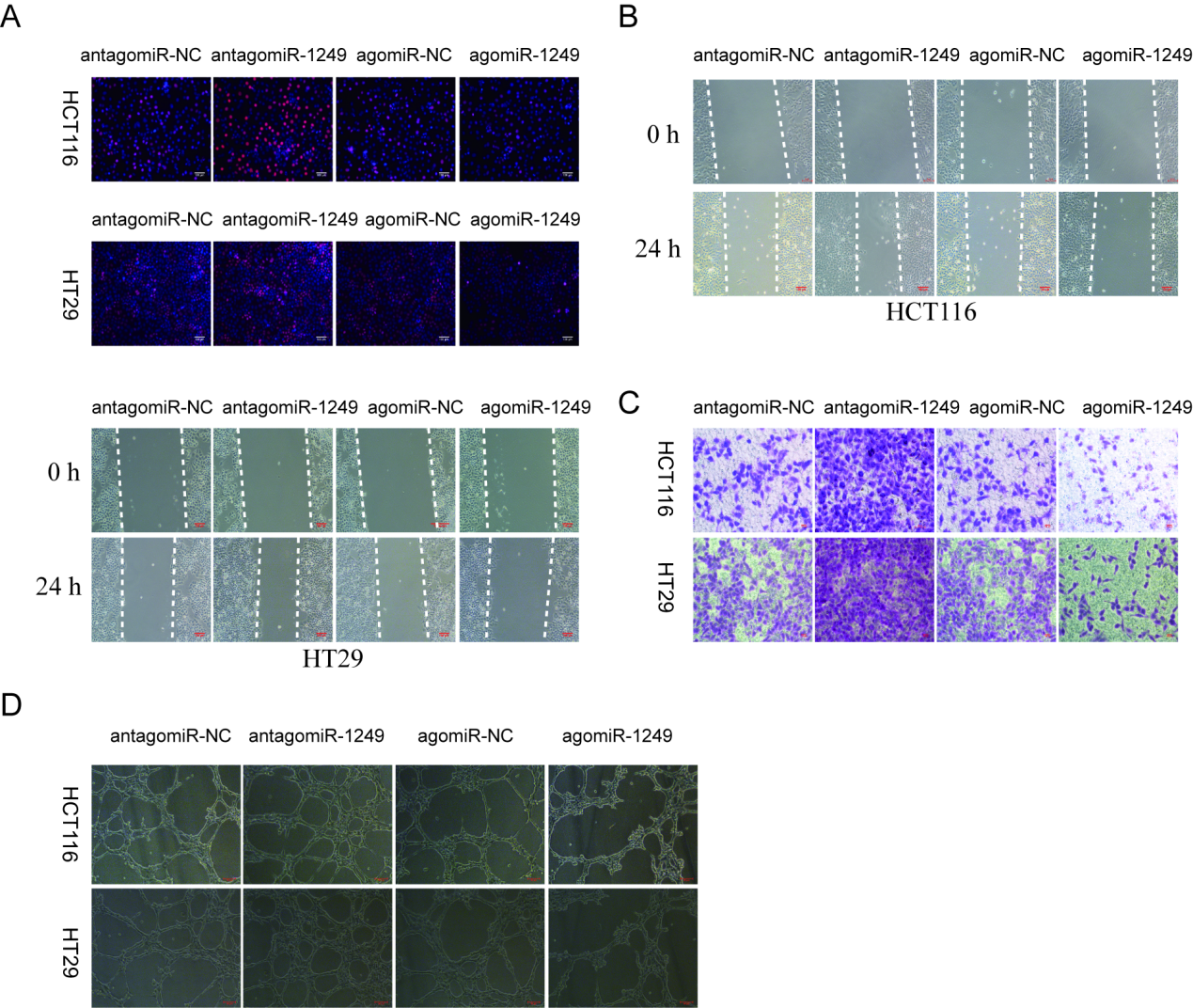


Fig S1


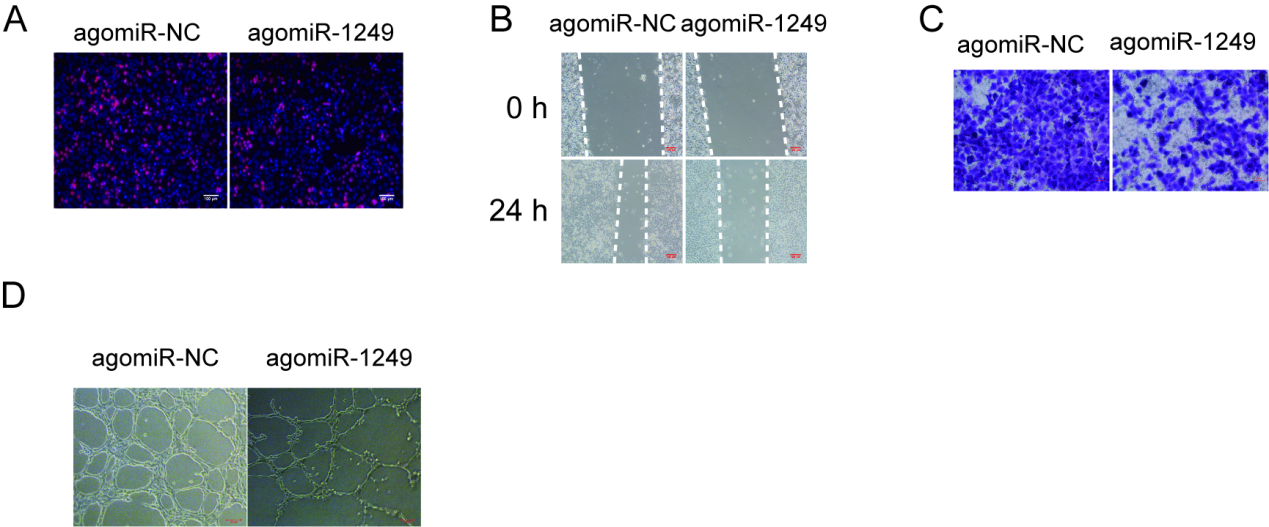


Fig S2

**
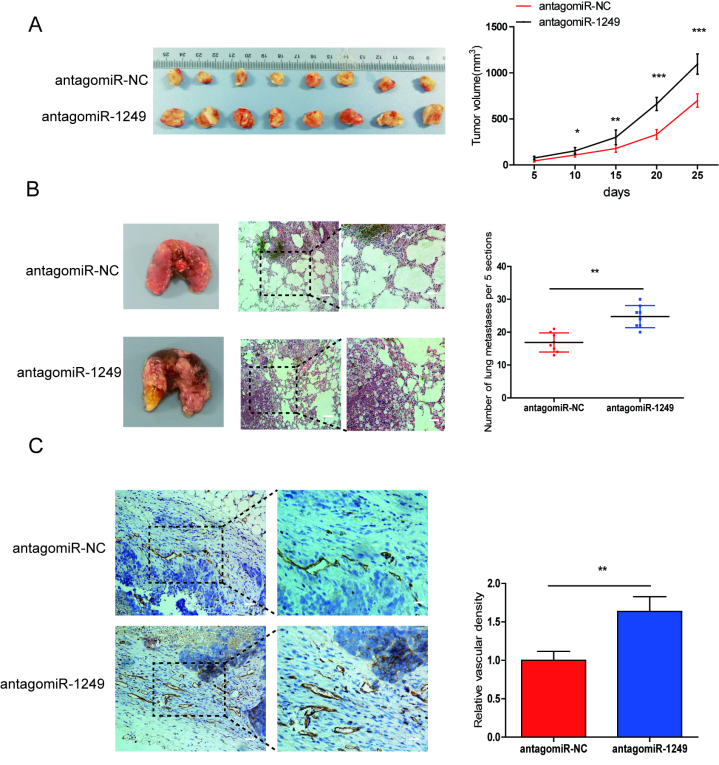
**

Fig S3


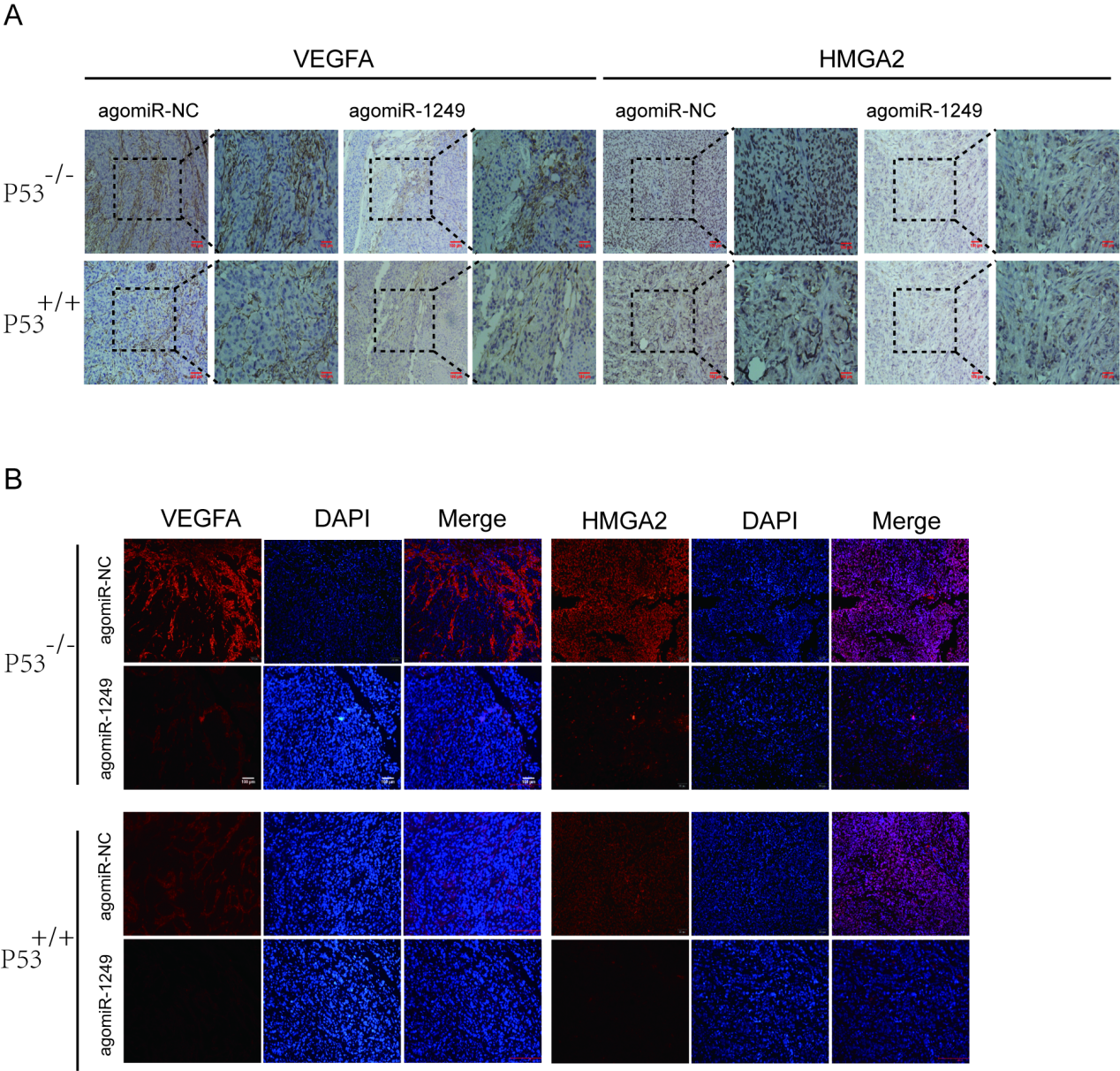


Fig S4


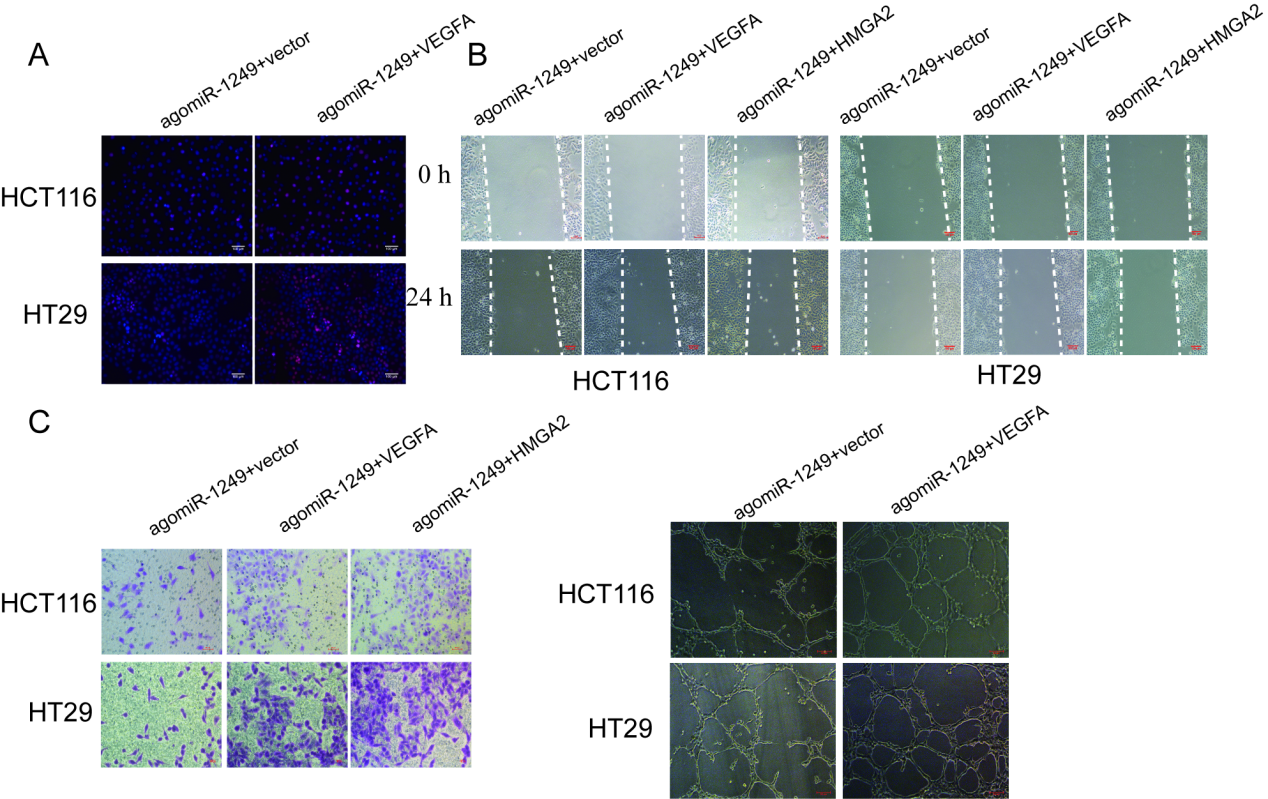


Fig S5
